# Hydrogen Sulphide Sequestration with Metallic Ions in Acidic Media Based on Chitosan/sEPDM/Polypropylene Composites Hollow Fiber Membranes System

**DOI:** 10.3390/membranes13030350

**Published:** 2023-03-17

**Authors:** Dumitru Pașcu, Aurelia Cristina Nechifor, Vlad-Alexandru Grosu, Ovidiu Cristian Oprea, Szidonia-Katalin Tanczos, Geani Teodor Man, Florina Dumitru, Alexandra Raluca Grosu, Gheorghe Nechifor

**Affiliations:** 1 Analytical Chemistry and Environmental Engineering Department, University Politehnica of Bucharest, 011061 Bucharest, Romania; 2 Department of Electronic Technology and Reliability, Faculty of Electronics, Telecommunications and Information Technology, University Politehnica of Bucharest, 061071 Bucharest, Romania; 3 Department of Inorganic Chemistry, Physical Chemistry and Electrochemistry, University Politehnica of Bucharest, 011061 Bucharest, Romania; 4 Department of Bioengineering, University Sapientia of Miercurea-Ciuc, 500104 Miercurea-Ciuc, Romania

**Keywords:** hydrogen sulphide sequestration, composite membranes, chitosan, sEPDM, polypropylene hollow fiber membrane, electronics waste

## Abstract

This paper presents the preparation and characterization of composite membranes based on chitosan (Chi), sulfonated ethylene–propylene–diene terpolymer (sEPDM), and polypropylene (PPy), and designed to capture hydrogen sulfide. The Chi/sEPDM/PPy composite membranes were prepared through controlled evaporation of a toluene dispersion layer of Chi:sEPDM 1;1, *w/w*, deposited by immersion and under a slight vacuum (100 mmHg) on a PPy hollow fiber support. The composite membranes were characterized morphologically, structurally, and thermally, but also from the point of view of their performance in the process of hydrogen sulfide sequestration in an acidic media solution with metallic ion content (Cu^2+^, Cd^2+^, Pb^2+^, and/or Zn^2+^). The operational parameters of the pertraction were the pH, pM, matrix gas flow rate, and composition. The results of pertraction from synthetic gases mixture (nitrogen, methane, carbon dioxide) indicated an efficient removal of hydrogen sulfide through the prepared composite membranes, as well as its immobilization as sulfides. The sequestration and the recuperative separation, as sulfides from an acid medium, of the hydrogen sulfide reached up to 96%, decreasing in the order: CuS > PbS > CdS > ZnS.

## 1. Introduction

Hydrogen sulfide (H_2_S) is a colorless gas with a specific smell, whose presence in the atmosphere is mainly of natural, but it can also appear in many industrial technologies, agriculture, and biotechnologies: wood and cellulose processing, the oil and gas industry, biodegradation of organic materials in waste recycling stations, fermentation processes, mining, and livestock farming [1,2].

Foul odor is one of the issues that is rarely addressed when discussing environmental pollution [3]. Of course, the multiple problems of air and water pollution in urban agglomerations, and even in isolated areas, leave on the margins the generation and fight against bad smells [4]. However, there are some concerns regarding this problem, especially when a bad smell is associated with the toxicity of some substances, as is the case with hydrogen sulfide [5,6]. Beyond the unpleasant sensation generated by hydrogen sulfide, it is also extremely toxic (because it combines with cytochrome iron and other essential compounds that contain iron in the cell), and long-term exposure of the population, even to very low concentrations, can cause serious diseases [7,8,9,10].

**Figure 1:**
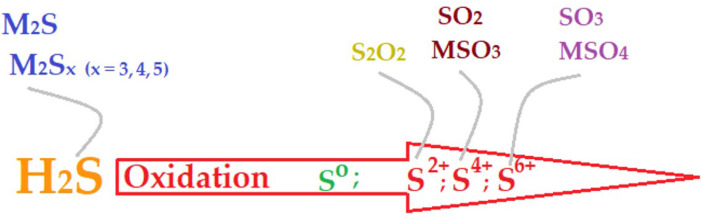
Schematic presentation of the removal, capture, and sequestration of hydrogen sulfide as metal sulfides or oxidation products: oxides, sulfites, sulfates.

From the point of view of chemical strategy (Figure 1, Table 1 and Figure S1 in the Supplementary Material), hydrogen sulfide can be captured in basic solutions of certain metals or with the help of their oxides (when the oxidation state of sulfur stays 2-) [11,12,13,14,15]. Another strategy consists in oxidizing sulfur from hydrogen sulfide to higher oxidation states (4+ or 6+) and recovering it as sulfuric acid or sulfites, but mostly as sulfuric acid and sulfate [16,17,18,19]. Biological degradation can also be an economical and effective means of hydrogen sulfide removal [20], while membrane or hybrid processes involving membranes have become more and more popular [21,22,23,24,25,26,27,28,29].

The hollow fiber type of membrane presents special operational advantages: a large surface area per volume unit, mounting options and provision of supply and stripping circuits, increased physical-chemical resistance, self-supporting structure, and ease of handling [30,31,32,33,34,35].

The elimination of hydrogen sulfide dispersed in the atmosphere is very uneconomical, and therefore it is recommended to remove it from the source [30]. Unfortunately, even this option is not always technically and economically possible [31].

On the other hand, there are also cases in which hydrogen sulfide is an impurity associated with other gases (domestic biogas installations, skunks, natural methane gas) in which an important component is also carbon dioxide, which must not be removed [31,32].

Studies on the separate retention of hydrogen sulfide without majorly affecting the composition of the treated carrier gas are relatively few, especially referring to small enclosures [33].

Due to their small volume, high selectivity, and low consumption of chemicals, membrane installations are a technique that can be considered for the purification of gases with a low content of hydrogen sulfide, both for the correction of the smell and for the reduction of toxicity and corrosion [34,35,36].

The membranes used for the removal, separation, concentration, analysis, and valorization of hydrogen sulfide are based on ionic polymers [37,38], biopolymers [39,40], organic [41], or inorganic composites [42]. Among the ionic polymers, sulfonated polyether ether ketone and sulfonated ethylene–propylene–diene terpolymer (sEPDM) are used [43], while among biopolymers, chitosan (Chi) is of particular interest, because it covers various membrane separations and spans the membranes for sensors [44,45,46,47,48,49,50].

Chitosan-ionic polymer composite membranes have recently been used as essential elements in fuel cells [51] and in the separation of certain metal ions [52].

This paper studied the recuperative separation (sequestration) of both hydrogen sulfide from the gaseous mixtures that it forms part of (traces), as well as some metallic cations that appear in the waste from the electronic and electrotechnical industry, as strongly acidic solutions. The central objective of the paper is motivated by the existence in urban waste processing platforms of both organic mass fermentation stations (biogas generators that must be purified from hydrogen sulfide in traces), as well as acidic attack installations for electrotechnical and electronic waste (generators of dilute solutions of metal ions, including Cu^2+^, Cd^2+^, Pb^2+^, and/or Zn^2+^, whose recovery is mandatory).

For the sequestration of hydrogen sulfide from the poor gaseous source phase (SP), pertraction through composite membranes based on chitosan (Chi), sulfonated ethylene–propylene–diene terpolymer (sEPDM), and polypropylene hollow fiber (PPy) in acidic receiving phases (RP) containing a metallic ion content (Cu^2+^, Cd^2+^, Pb^2+^, and/or Zn^2+^) was carried out.

## 2. Materials and Methods

### 2.1. Reagents and Materials

The materials used in the present work were of analytical purity. They were purchased from Merck (Merck KGaA, Darmstadt, Germany)—sodium sulfide (Na_2_S) [78.0452 g/mol (anhydrous)], hydrogen sulfide, sodium hydroxide, nitric acid, and hydrochloric acid.

Cu(NO_3_)_2_·3H_2_O, Cd(NO_3_)_2_·4H_2_O, Pb(NO_3_)_2_, Zn(NO_3_)_2_, NaCl, chitosan, and glacial acetic acid (analytical grade, Sigma-Aldrich Chemie GmbH, Steinheim, Germany) were used in the studies. NaOH pellets, H_2_SO_4_ (96%), HCl (35%), HNO_3_ (62%) ultrapure, and NH_4_OH 25% (analytical grade) were purchased from Merck KGaA Darmstadt, Germany.

Ultrapure waters were used for preparing the feeding solutions in every case. The purified water, characterized by a 18.2 µS/cm conductivity, was obtained with a RO Millipore system (Milli-Q^®^ Direct 8 RO Water Purification System, Merck KGaA, Darmstadt, Germany).

The tubular dialysis membranes were from Visking (Medicell Membranes Ltd., London, UK). An MQuant^®^ sulfide test (Merck Millipore from Merck KGaA, Darmstadt, Germany), Sulfide Test photometric, Spectroquant^®^ was used (Merck KGaA, Darmstadt, Germany).

The polymers used to obtain composite membranes were chitosan (Chi) (Sigma-Aldrich Chemie GmbH, Steinheim, Germany) and sulfonated ethylene–propylene–diene terpolymer (sEPDM), which have recently been used in our research group for ionic and molecular separations [53]. Their main characteristics are given in Table 2.

The hollow polypropylene fibers used as membrane support (PPM) were provided by GOST (GOST Ltd., Perugia, Italy) and their characteristics were presented in detail in our previous works [54].

### 2.2. Procedures

#### 2.2.1. Preparation of Composite Membranes (Chi/sEPDM/PPy)

A dispersion containing a 10 g/L mixture of chitosan polymers (Chi) and sulfonated ethylene–propylene–diene terpolymer (sEPDM) (1:1; *w*/*w*) in toluene was prepared by dispersing 5 g of chitosan powder in a liter of toluene solution of sulfonated ethylene–propylene–diene terpolymer (5 g/L).

**Figure 2:**
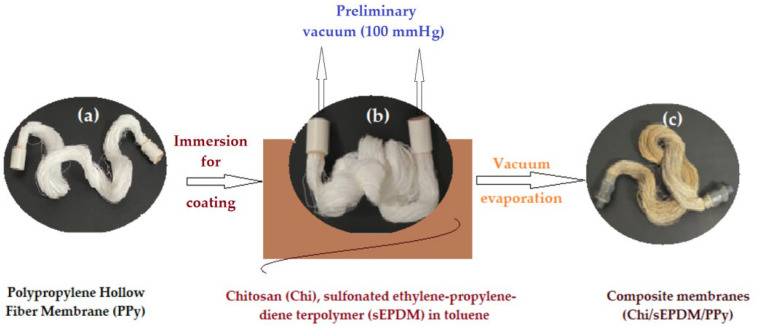
Schematic presentation of the Chi/sEPDM/PPy composite membrane production process: (**a**) virgin polypropylene hollow fiber membranes; (**b**) membranes immersed in the chitosan–sEPDM toluene dispersion bath; (**c**) composite membranes (Chi/sEPDM/PPy).

Hollow polypropylene fiber membranes (PPy) (Figure 2) were assembled so that they could be mounted in a pertraction module, which ensured a mass transfer surface of 1.0 m^2^. The mass of the fiber assembly was determined on an analytical balance (Figure 2a), two preliminary vacuum (100 mmHg) extensions were attached to the ends of the assembly, and immersed for 30 min in a vessel containing the chitosan dispersion in 2.0 L toluene solution of Chi/sEPDM (Figure 2b), in order to cover the fibers with a film of composite polymer dispersion. After 30 min, the vacuum source was removed, and the membrane bundle was released from the extension, to move on to the operation of removing toluene from the adherent Chi/sEPDM film (Figure 2c) and to generate a new structure by phase inversion induced by controlled evaporation of the Chi/sEPDM/PPy composite membrane. For these processes, the membrane bundle was placed in a vacuum oven for drying at 60 °C, for two hours. The laboratory ambient conditions for the production of composite membranes were temperature 25 ± 1 °C, atmospheric pressure 761 ± 1 mmHg, and a humidity of 50 ± 3%.

The assembly of membranes covered with the composite membrane was placed into a desiccator, to cool down to room temperature, and then it was weighed on an analytical balance, in order to evaluate the amount of polymer in the fibers. If the mass did not remain constant, the assembly of composite fibers was reintroduced into the vacuum oven for 10 min and the operation was repeated until the mass became constant. Two sealing elements were attached to the ends of the assembly of fibers, by immobilization with acrylic polymer (Figure 2c). In parallel, the Chi/sEPDM composite membranes and the sEPDM membranes required for characterization as control samples were prepared [55].

**Figure 3:**
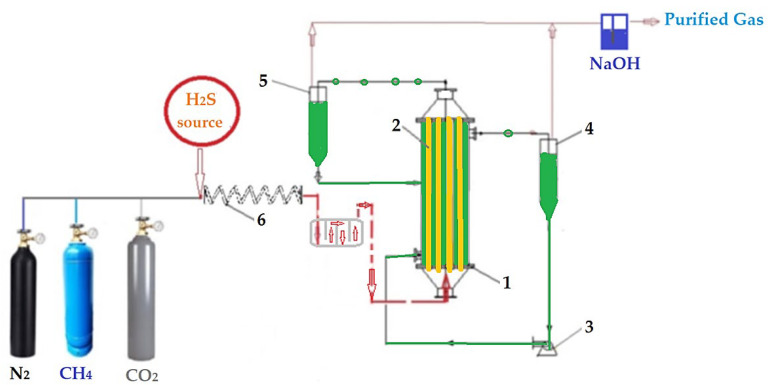
Schematic presentation of the laboratory installation for hydrogen sulfide sequestration from a gaseous mixture: 1—membrane contactor; 2—composite hollow fiber membranes; 3—pump for metal ions acidic solutions; 4 and 5—gas-liquid separator; 6—homogenization.

The composite membrane bundle was mounted in a pertraction module, thus obtaining a membrane contactor analogous to a tubular heat exchanger, similar to those described extensively in our previous works [56,57,58]. This module was located in the working installation (Figure 3).

#### 2.2.2. Pertraction of Hydrogen Sulfide with Composite Membranes

The solutions (Cu^2+^, Zn^2+^ or/and Cd^2+^) were prepared from the available reagents without any restrictions, but the aqueous solutions containing Pb^2+^ required precautions, therefore the corresponding nitrates were used.

The separation tests were performed with 5 × 10^−7^–10^−3^ mol/L solutions of Cu(NO_3_)_2_, Cd(NO_3_)_2_, Zn(NO_3_)_2_, and Pb(NO_3_)_2_ obtained in ultrapure water [59].

To realize the pertraction, experiments for the hydrogen sulfide sequestration from gaseous mixture were performed in installations with the tubular configuration module presented in Figure 3. The central element of the installation was the traction module (1) in which the composite membranes were fixed. Five liters of receiving solution with the required composition (pH and concentration of metal ions) was transported by the pump (3) through the outside of the membrane fibers, with a variable flow rate of 100–500 mL/min. The source phase consisting of the gaseous mixture containing hydrogen sulfide was circulated through the capillary fibers, with a flow rate of 2–20 L/min. The target gas mixtures had a matrix of nitrogen, methane, carbon dioxide (simulating domestic applications), and the target concentration of hydrogen sulfide of 20–60 ppm, but the study was extended up to 120 ppm.

The residual gas was passed through gas–liquid separators (Figure 3, elements marked as 4 and 5) and upon evacuation it was passed through a sodium hydroxide trap. The composition of the source gaseous phase was obtained by dosing the matrix gas (nitrogen, methane, or carbon dioxide) by means of a system of reducers and flowmeters, which allowed the regulation of the flow rate, into which an appropriate amount of hydrogen sulfide was injected. To homogenize the gas mixture, a 10 m long glass capillary coil and a detention vessel were used. The operational parameters of the pertraction were the pH, pM, the flow rate of the matrix gas, and its composition.

These operational parameters (pH, pM, the flow, and the composition of gaseous mixture) were specified for each individual experiment, and each type of experiment was repeated three to five times (from case to case) to assess the maximum accuracy.

The gas composition was determined by means of a specific sensor, after the homogenization coil and before the sodium hydroxide trap (Figure 3). Validation of the results was also performed by analyzing the concentration of sulfide ions in the trap with sodium hydroxide [60].

The pertraction efficiency (*PE*%) for the species of interest (hydrogen sulfide) using the concentration of the solutions [61] was calculated as follows, Equation (1):

(1) $$PE\left(\%\right)=\frac{\left(c_{0}-c_{f}\right)}{c_{0}}\times 100$$

*c_f_* being the final concentration of the solute (hydrogen sulfide) and *c*_0_ the initial concentration of solute (hydrogen sulfide).

The measurements were independently validated using an Oldham MX 21 gas detector (MX 21 Plus Multigas, Arras, France) equipped with electrochemical sensors, and a H_2_S Model 3000RS Analyzer (MultiLab LLC, Bucharest, Romania) and analytical rapid tests [1,62].

Parallel determination of the free metallic ions in the receiving phase was performed with atomic absorption spectrometry.

### 2.3. Equipment

The scanning microscopy studies, SEM and HR-SEM, were performed using a Hitachi S4500 system (Hitachi High–Technologies Europe GmbH, Mannheim, Germany) [63].

Thermal analysis (TG-DSC) was performed with a STA 449C Jupiter apparatus, from Netzsch (NETZSCH-Gerätebau GmbH, Selb, Germany). Each sample was weighed as approximatively 10 mg. The samples were placed in an open alumina crucible and heated up to 900 °C with a 10 K∙min^−1^ rate, under a flow of 50 mL∙min^−1^ dried air. As a reference, we used an empty alumina crucible. The evolved gases were analyzed with a FTIR Tensor 27 from Bruker (Bruker Co., Ettlingen, Germany), equipped with a thermostat gas cell [64].

The UV–Vis analyses of the solutions were carried out on a Spectrophotometer CamSpec M550 (Spectronic CamSpec Ltd., Leeds, UK) [65].

The pH of the medium was tested with a combined selective electrode (HI 4107, Hanna Instruments Ltd., Leighton Buzzard, UK) and a multi-parameter system (HI 5522, Hanna Instruments Ltd., Leighton Buzzard, UK) [66].

To assess and validate the content of metal ions, an atomic absorption spectrometer AAnalyst 400 AA Spectrometer (Perkin Elmer Inc., Shelton, CT, USA) with a single-element hollow-cathode lamp was used, driven by WinLab32–AA software (Perkin Elmer Inc., Shelton, CT, USA) [67].

## 3. Results and Discussion

The recuperative separation addressed in this study had as objective the purification of some common gases (nitrogen, methane, and carbon dioxide) from impure hydrogen sulfide, by sequestering it as usable sulfides from acid solutions with a low content of metal ions (Cu^2+^, Cd^2+^, Pb^2+^, and/or Zn^2+^), resulting as waste from the electronic and electrotechnical industry, and according to the chemical reactions described by Equations (2)–(5):

Cu^2+^ + H_2_S + 2H_2_O ⇌ CuS_(s)_ + 2H_3_O^+^ (2)

Zn^2+^ + H_2_S + 2H_2_O ⇌ ZnS_(s)_ + 2H_3_O^+^ (3)

Pb^2+^ + H_2_S + 2H_2_O ⇌ PbS_(s)_ + 2H_3_O^+^ (4)

Cd^2+^ + H_2_S + 2H_2_O ⇌ CdS_(s)_ + 2H_3_O^+^ (5)

The Chi/sEPDM/PPy composite membrane used for the separation of hydrogen sulfide was prepared by coating the tubular polypropylene fiber through immersion in a chitosan dispersion in a toluene solution of sEPDM, followed by controlled evaporation, and it was morphologically and structurally characterized by scanning electron microscopy (SEM), Fourier transform infrared spectroscopy (FTIR), energy-dispersive spectroscopy analysis (EDAX), thermal analysis (TG, DSC), thermal analysis coupled with chromatography, and infrared analysis.

The performance of the pertraction process was evaluated by varying the operational parameters pH, pM (for the receiving phase), matrix gas flow rate, and the composition (for the source phase).

### 3.1. Morphological and Structural Membrane and Membrane Material Characteristics

#### 3.1.1. Scanning Electron Microscopy (SEM)

Chi/sEPDM/PPy composite membrane samples with a length of 3 cm were fractured in liquid nitrogen and metallized with a superficial layer of gold, to allow the surface and the section of the membranes to be analyzed by scanning electron microscopy (SEM) coupled with energy-dispersive X-ray analysis (EDAX), with a Hitachi S4500 system.

**Figure 4:**
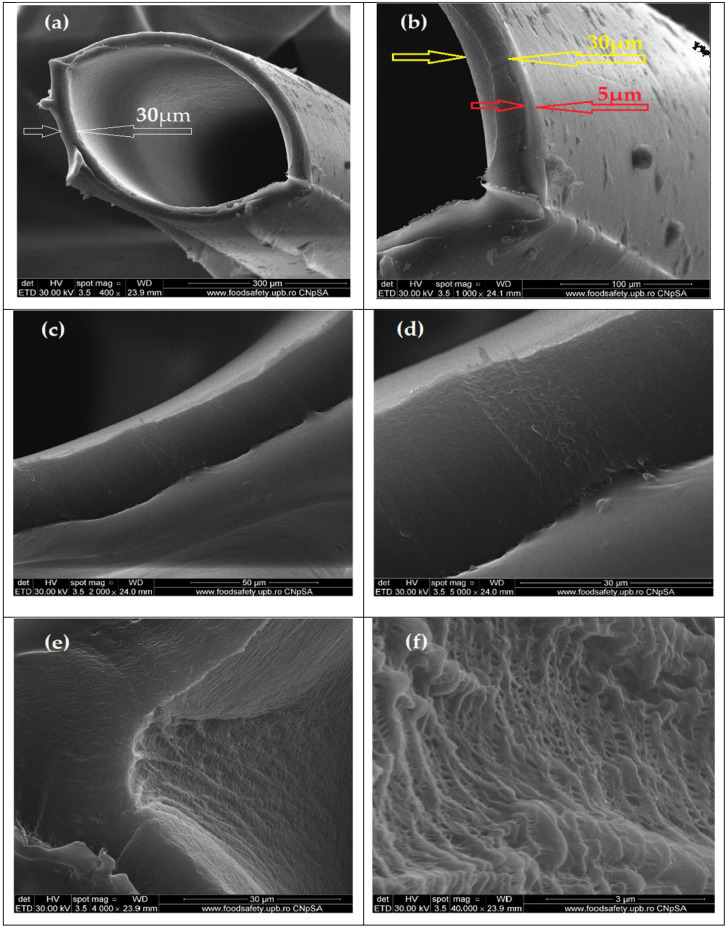
Scanning electron microscopy (SEM) images for the Chi/sEPDM/PPy composite membranes: (**a**) cross-section 400×; (**b**) cross-section 1000×; (**c**) wall detail 2000×; (**d**) wall detail 5000×; (**e**) inside aspect of wall 4000×; and (**f**) inside aspect of wall 40,000×.

The composite membrane morphology (Chi/sEPDM/PPy) is shown in Figure 4.

Thus, Figure 4a shows an image of the membrane profile examined from the right side, at a low magnification of 400×, in which the outer and inner diameters of the fiber can be distinguished at around 300 µm. In Figure 4b, at a magnitude of 1000×, one can see the uniformity of the superficial deposition of chitosan and sulfonated ethylene-propylene-diene terpolymer, which formed a consistent active layer (approx. 5 µm). The thickness of almost 30 µm (Figure 4a,b) of the wall of the polypropylene fiber can be also evaluated, in Figure 4c,d, taken at 2000× and 5000× magnifications, respectively. Looking from the right side of the inside of the polypropylene fiber, as shown in Figure 4a, the characteristics of the size and distribution of the pores at magnitudes of 4000× (Figure 4e) and 40,000× (Figure 4f) are highlighted, which confirmed the results presented in our previous works [57,58,59].

**Figure 5:**
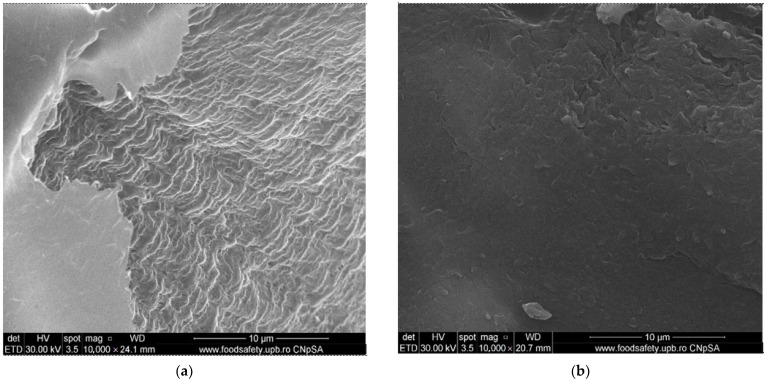

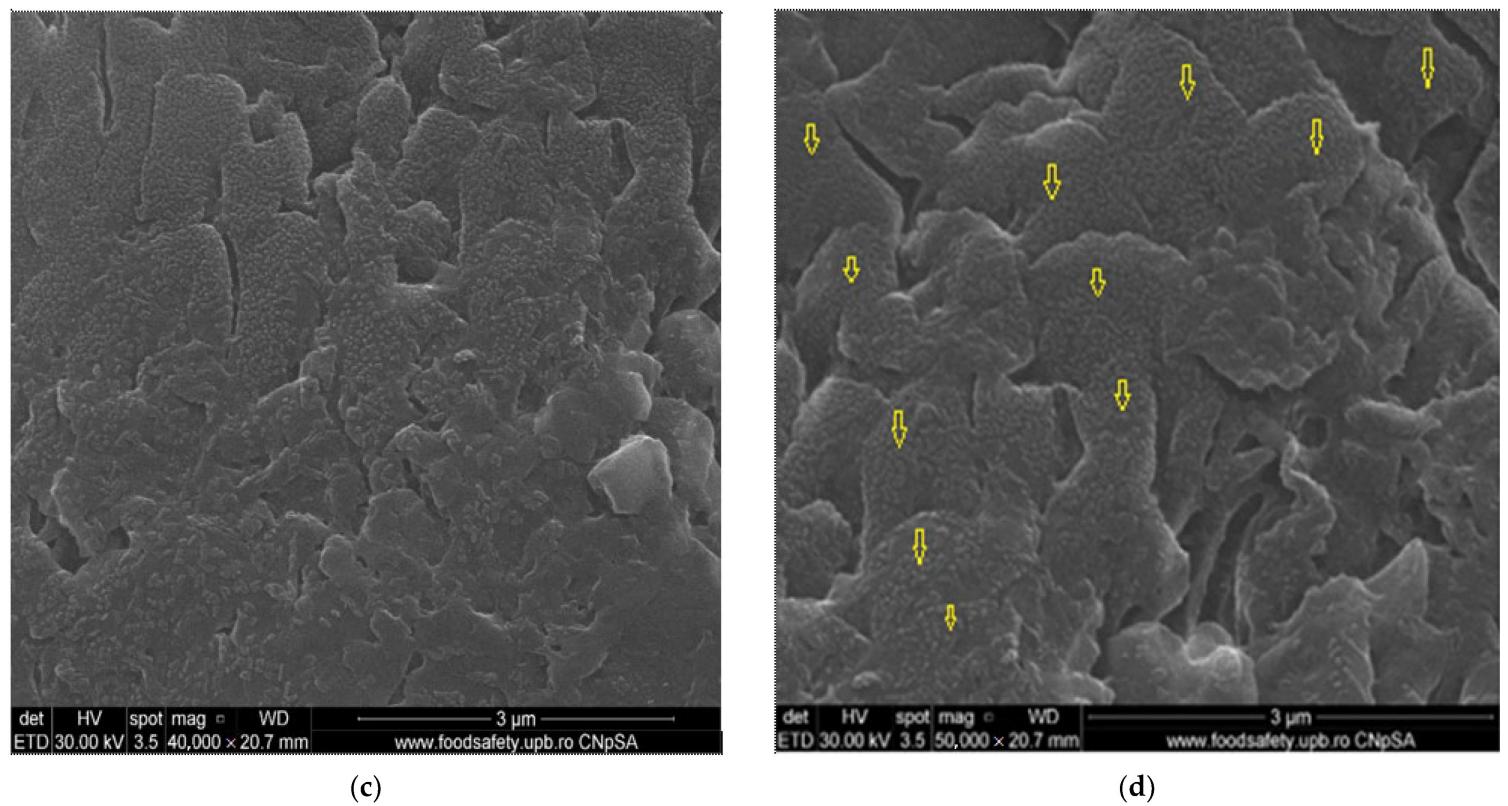
Top surface scanning electron microscopy (SEM) images for the Chi/sEPDM/PPy composite membranes: (**a**) 10,000×; (**b**) 10,000×; (**c**) 40,000×; and (**d**) 50,000×.

**Figure 6:**
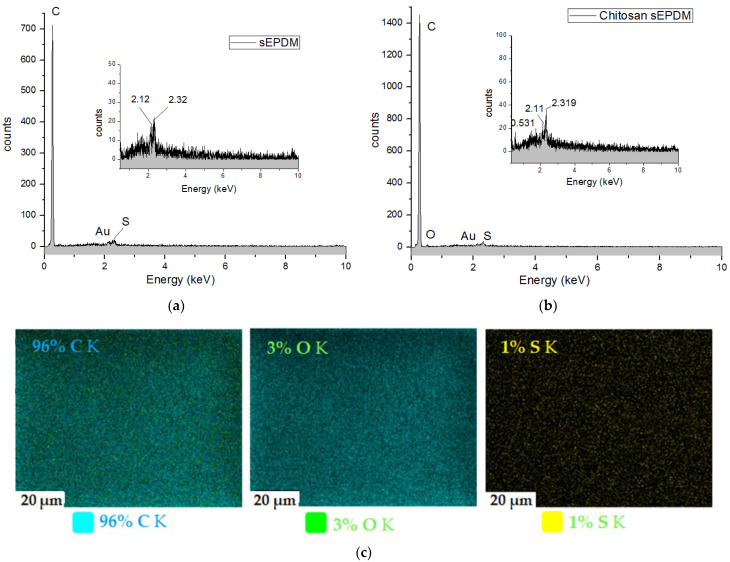

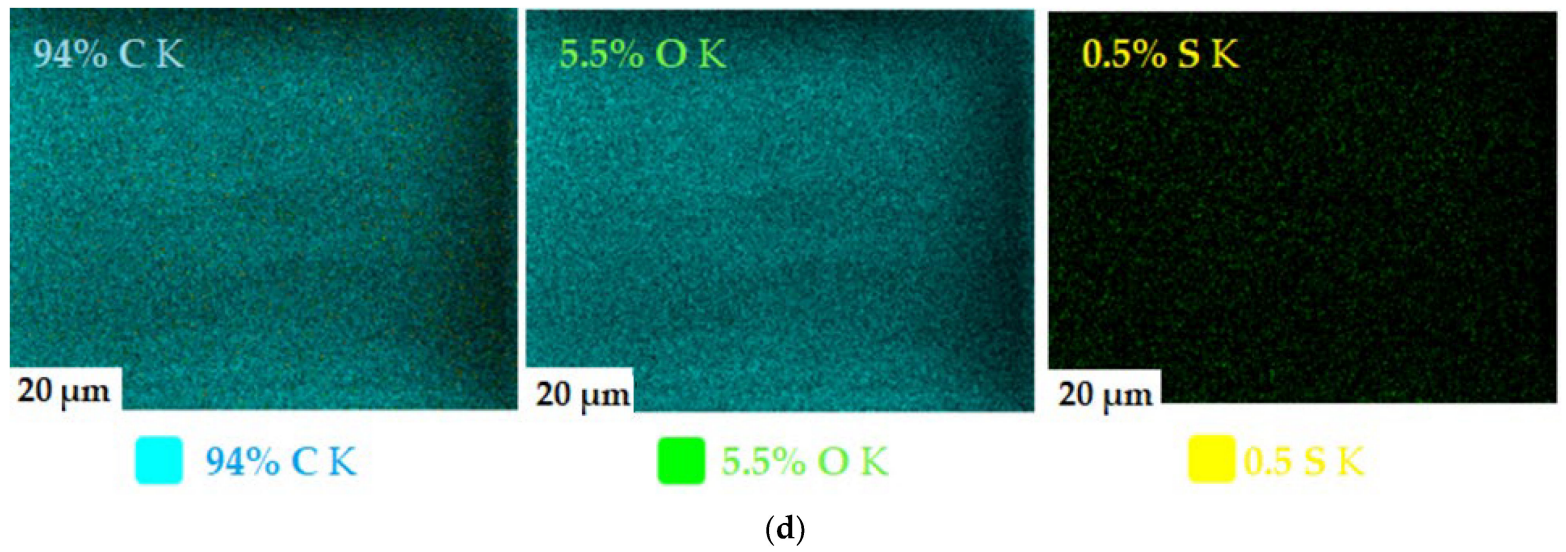
Energy-dispersive spectroscopy analysis (EDAX) diagram for the membrane materials: sEPDM (**a**); Chi/sEPDM (**b**); and elemental maps: sEPDM (**c**); Chi/sEPDM (**d**).

In Figure 5, attention is focused on the active membrane layer consisting of chitosan and sulfonated ethylene–propylene–diene terpolymer. Thus, the active layer presented in the left part of the image in Figure 6a (using a magnitude of 10,000×) was swept to the left, obtaining an image with a wider perspective (Figure 5b, magnitude 10,000×) that highlights the Chi/sEPDM composite surface. By careful examination of the details of the surface of the active layer of chitosan and sulfonated ethylene–propylene–diene terpolymer at the magnitude of 40,000× (Figure 5c), the surface of the composite membrane has a platelet-like (scaly) appearance, in which the continuous phase (base of the plate) is sEPDM, and the nanoparticles are made of chitosan (marked with yellow arrows—Figure 5d, magnitude 50,000×).

The data provided by scanning electron microscopy (SEM) (Figure 5) were complemented by an energy-dispersive spectroscopy analysis (EDAX) diagram (Figure 6) for the top surface membrane materials: sulfonated ethylene–propylene–diene terpolymer (sEPDM) (Figure 6a) and chitosan (Chi)–sulfonated ethylene-propylene–diene terpolymer (Chi–sEPDM) (Figure 6b). However, in the EDAX spectra, only the carbon (C), oxygen (O), and sulfur (S) atoms appear distinctly, since the nitrogen atoms could not be highlighted due to the working technique. The structure of the active layer was also examined using spectroscopy and microscopy in the infrared range.

In Figure 6c,d, elemental distribution maps can be observed. The surface carbon concentration was higher in the sEPDM membrane compared to the Chi/sEPDM composite one, while the value of the oxygen concentration was reversed (the contribution of chitosan atoms being obvious). The surface concentration of sulfur was lower in the case of the composite membrane (it was reduced by half). However, given the errors in the analysis method (C error of 3.07%; O error of 29.5%, and S error of 62.31%), but also the fact that this examination was local, the results must be considered qualitative and require confirmation using alternative methods.

#### 3.1.2. Fourier Transform InfraRed Spectroscopy (FTIR) Membrane Characteristics

The data obtained by elemental analysis (EDAX) required a study in the infrared domain, both spectrally (FTIR) and by interference reflection microscopy (IRM), to complete the structural information and the surface composition of the composite membranes compared to the prepared control membranes.

**Figure 7:**
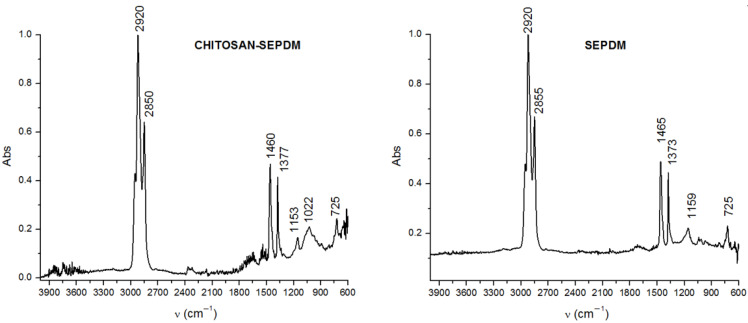
Fourier transform infrared spectra for the composite membranes: sEPDM and Chi/sEPDM composite membrane.

FTIR spectra were obtained for the control membranes: sulfonated ethylene–propylene–diene terpolymer membranes (sEPDM) and chitosan/sulfonated ethylene–propylene–diene terpolymer composite membranes (Chi/sEPDM) (Figure 7).

The FTIR spectrum of sEPDM (Figure 7) has specific absorption bands, due to the valence vibration of the C–H bonds located at 2855 cm^−1^ and 2920 cm^−1^; those at 1465 cm^−1^, where absorptions of medium intensity appear due to the vibrations of deformation of the methylene groups (–CH_2_−); those at 1373 cm^−1^, where bands of medium intensity appear due to the symmetric deformation vibration of the methyl groups (–CH_3_); and those with the value of 725 cm^−1^ due to deformation vibrations outside the plane of the C–H bonds.

The FTIR spectrum of the Chi/sEPDM composite membrane (Figure 7) was dominated by the adsorption bands of sEPDM, but at slightly shifted values compared to the pure sEPDM, due to interactions with chitosan. However, low-intensity adsorption bands specific to some functional groups of chitosan can be noted (3600 cm^−1^–3750 cm^−1^ extended hydrogen bonds, and strong broad band at 1022 cm^−1^ corresponding to C-O stretching from Chi).

The absorption band localized at 1154 cm^−1^ can be attributed either to CH (propylene) or to the O=S=O groups.

Unfortunately, the spectra obtained (Figure 7) did not show specific bands that could be used in the examination by reflective microscopy in the infrared range (MRI), and therefore, for the examination of the chitosan/sulfonated ethylene–propylene–diene terpolymer surface layer of the composite membrane, we chose a wavenumber from each specific interval of the domain: 3345 cm^−1^, 1385 cm^−1^, 1050 cm^−1^, and 728 cm^−1^.

**Figure 8:**
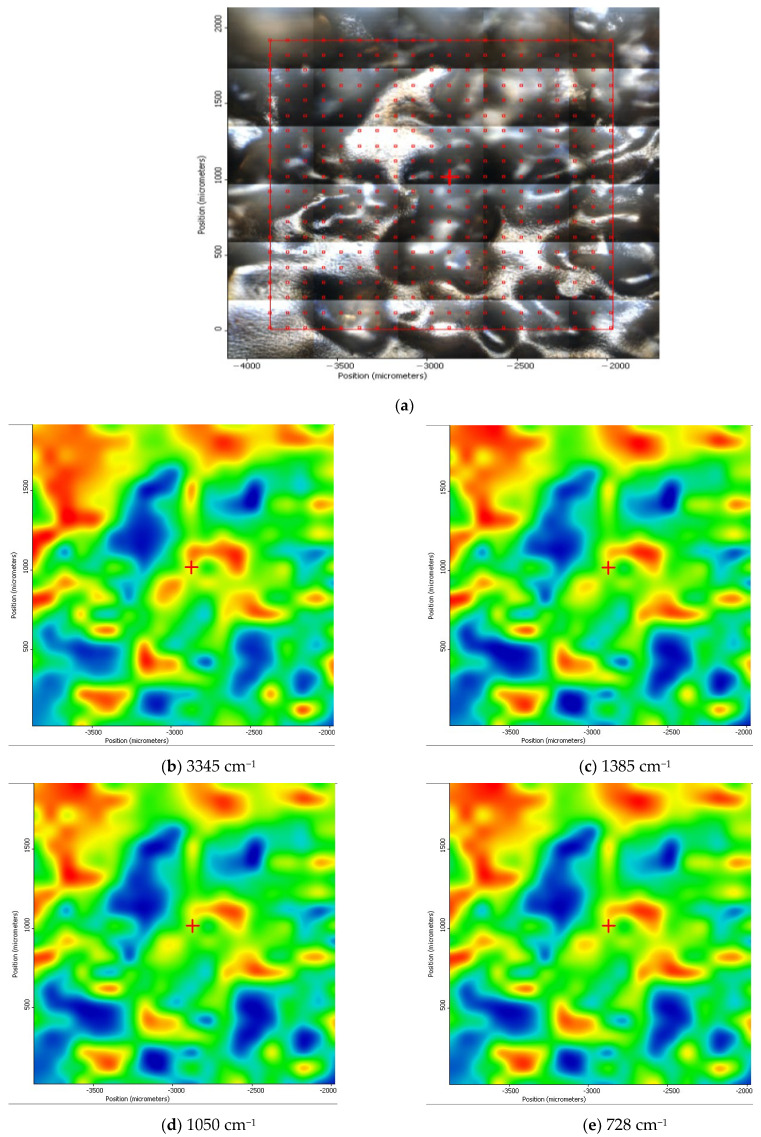

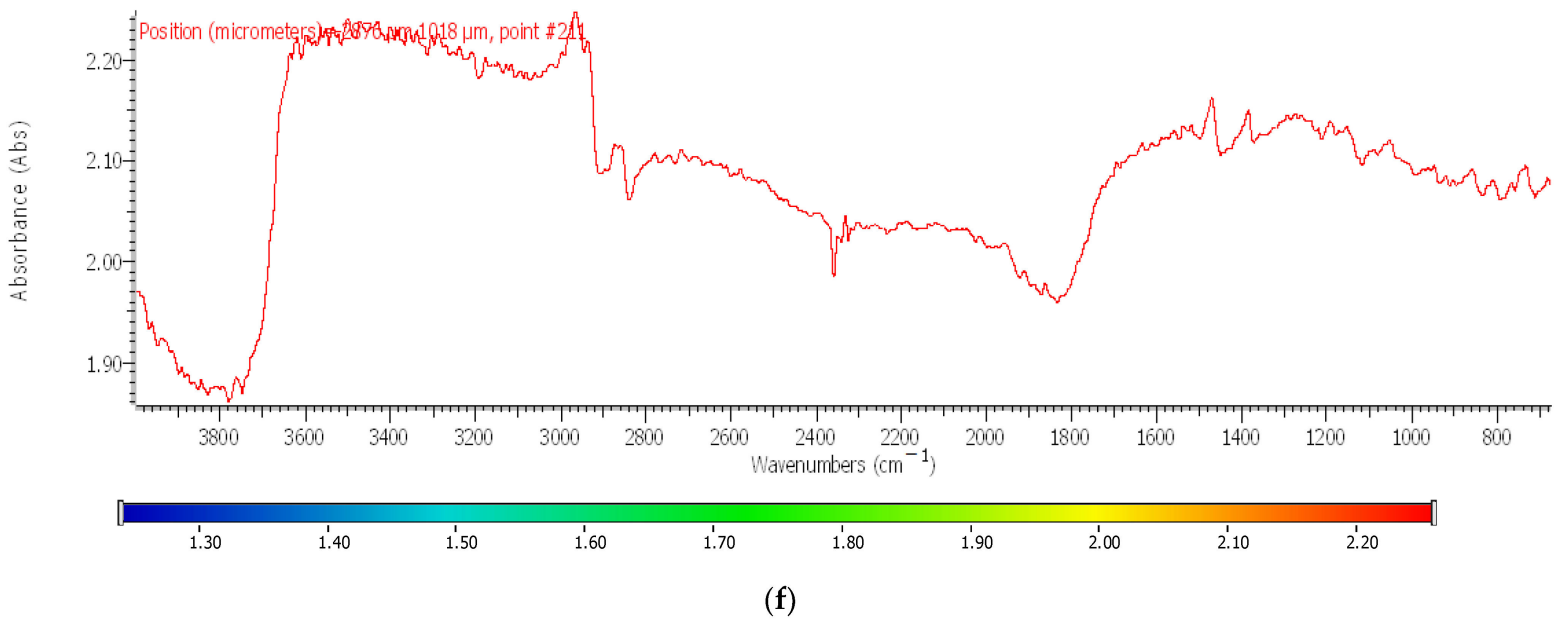
Video-images (**a**), the 2HD-IR obtained maps at the specific wave number (**b**–**e**); and infrared associated spectrum and color scales (**f**); for Chi/sEPDM composite membrane.

The HD-IR maps obtained for the area of the composite membrane (Figure 8a) showed a remarkable spectral uniformity (Figure 8b–e), embodied in the formal overall spectrum shown in Figure 8f. These results show that the superficial layer examined was almost entirely formed by sEPDM, which fully covered the composite membrane, including the chitosan nanoparticles highlighted in Figure 5c,d. The upper layer of sEPDM that covered the composite membrane could support the pertraction process, considering that its absence can cause the implosion of chitosan particles.

**Figure 9:**
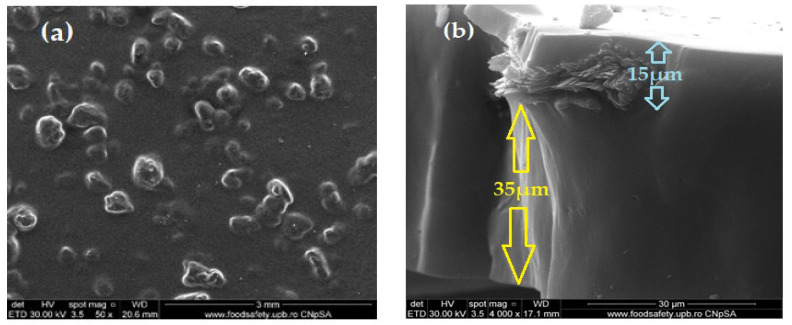
Scanning electron microscopy (SEM) images for the Chi/sEPDM composite membrane: top surface (**a**); and cross-section (**b**).

We observed a phenomenon, which it is still under study, when testing the Chi/sEPDM composite membrane, whereby chitosan aggregates subjected to osmotic pressure during the pertraction process accumulated water, potentially degrading the membrane (Figure 9).

#### 3.1.3. Thermal Characteristics of the Prepared Test Membranes

A complex thermal analysis was carried out, both to monitor the thermal behavior for use of the composite membrane in processes at temperatures higher than the ambient temperature (the range up to 200 °C being the target), but also to confirm the composition of the Chi/sEPDM composite membrane (the range of interest being the one above 200 °C). The composition of the membrane was determined through gas chromatographic analysis coupled with infrared spectrometry analysis of the combustion gases (up to 800 °C).

**Figure 10:**
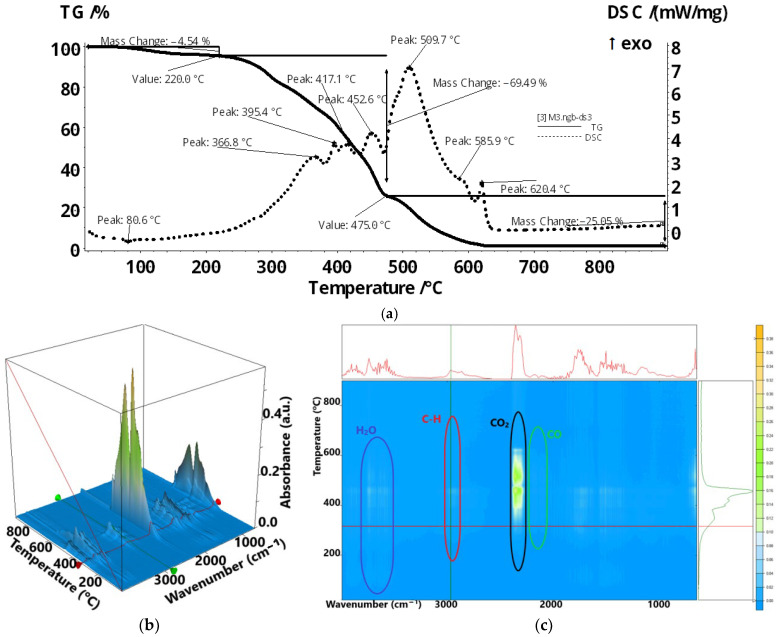
Thermal characteristics of the sEPDM membrane: (**a**) thermal diagram; (**b**) 3D complex analysis; (**c**) 2D complex analysis. (Reprinted from Ref. [55]).

The Chi/sEPDM composite membrane sample lost 4.54% up to 220 °C (Figure 10a), mostly water molecules and some traces of SO_2_, as indicated by the FTIR spectra (Figure 10b,c). The main degradation processes took place between 220–475 °C, when a series of exothermic effects were observed on the DSC curve, indicating multiple oxidation reactions. Most of the gaseous products, CO, H_2_O, hydrocarbon fragments, and SO_2_, were removed in this interval. The recorded mass loss was 69.49%. After 475 °C, the residual carbon mass was burned, with the main degradation product identified by FTIR being CO_2_.

Figure S2 (in the Supplementary Material) shows the released gases, identified as traces, during the thermal decomposition of the sample, through examination at specific wavenumbers: sulfur dioxide (SO_2_) at 1367 cm^−1^ (Figure S2a), carbon dioxide (CO_2_) at 2355 cm^−1^ (Figure S2b), hydrocarbons at 2964 cm^−1^ (Figure S2c), and water (H_2_O) at 3566 cm^−1^ (Figure S2d).

### 3.2. The Pertraction Performance for the Hydrogen Sulfur Separation with Prepared Composite Membranes (Chi/sEPDM/PPy)

The removal of hydrogen sulfide from various gaseous mixtures (air, biogas, natural methane gas) can be performed both by established classical methods [68] such as adsorption on iron sponge or iron oxide pellets, activated carbon, water scrubbing, NaOH scrubbing, and biological removal on a filter bed, as well as also using some modern ones: chemical oxidation, photo-oxidation, electrochemical oxidation, catalytic oxidation, extraction, and pertraction [69].

In what follows, we present the results of the hydrogen sulfide retention experiments with a concentration between 20 and 120 ppm, from synthetically produced gas mixtures (nitrogen, methane, and carbon dioxide), which reasonably simulated the situations encountered in air pollution (farms, tourist resorts, and household garbage depots), the impurity of natural methane, and also the biosynthesis gases that contain these components in various concentrations [70].

The objective of the study was the removal of hydrogen sulfide impurities from the source synthetic gaseous phases by sequestration in acidic solutions containing metal ions (Cu^2+^, Zn^2+^, Cd^2+^, Pb^2+^), so that metal cations were recovered as sulfides formed by the sequestration of hydrogen sulfide. The selected metal ions are common components of electronic and electrotechnical industry waste and are found in acid leaching solutions [71,72].

The hydrogen sulfide sequestration process was carried out by pertraction with chitosan/sulfonated ethylene-propylene-diene terpolymer/polypropylene hollow fiber composite membranes (Chi/sEPDM/PPy), aiming at the following parameters of the process: the concentration and flow of the source gas phase, and the pH (acidity) and pM (concentration of metal ions) of the receiving ionic aqueous phase. Following the obtained results, a mechanism is proposed for the process of hydrogen sulfide pertraction from gaseous phases using acidic aqueous solutions containing metal ions.

The parameters of each experimental test for the membrane systems are specified in Table 3.

#### 3.2.1. The Influence of the pM of the Receiving Phase on Hydrogen Sulfide Pertraction through Chi/sEPDM/PPy–CM from Synthetic Gas Mixture

In order to assess the influence of the concentration of metal ions in the receiving phase on the efficiency of hydrogen sulfide pertraction from a synthetic mixture of source gases, the installation shown in Figure 3 was used with the parameters of the membrane phases specified in Table 3, experimental test I.

**Figure 11:**
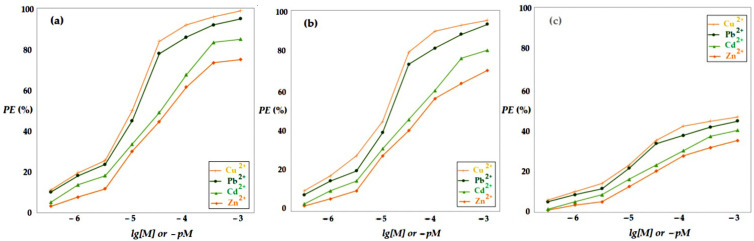
Hydrogen sulfide pertraction efficiency (*PE*%) vs. pM for 20 ppm H_2_S in gas mixture: nitrogen (**a**); methane (**b**), and carbon dioxide (**c**).

The obtained results are presented in Figure 11 and indicate an increase in the hydrogen sulfide extraction efficiency with the increase in concentration of metal ions in the receiving phase.

The matrix of the gas mixture containing hydrogen sulfide influenced the extraction efficiency, being significantly higher for nitrogen and methane than for carbon dioxide, over the entire concentration range, for all four cations.

The efficiency of the extraction depending on the composition of the receiving phase depended on the nature of the metal ion in the receiving solution, decreasing in the following order: Cu^2+^ > Pb^2+^ > Cd^2+^ > Zn^2+^.

The extraction efficiency when the matrix was carbon dioxide was reduced almost by half for all the working systems studied, which indicated competition at the level of the gas solubilization in the composite membrane. Therefore, the proposed hydrogen sulfide capture system requires special attention when chemical species appear in the gas mixture, which interact with the components of the chitosan/sulfonated ethylene-propylene-diene terpolymer/polypropylene hollow fiber composite membrane.

From the point of view of the metal ion concentration in the solution, a value close to 10^−3^ mol/L is preferable. This assumes that all the solutions that come from the processing of waste from the electronic and electrotechnical industry can be utilized without changing the pH, but they must have an average content of cations above 10^−4^ mol/L.

#### 3.2.2. The Influence of the pH of the Receiving Phase on Hydrogen Sulfide Pertraction through a Chi/sEPDM/PPy Composite Membrane from Synthetic Gas Mixture

Chi/sEPDM/PPy composite membranes for the sequestration of hydrogen sulfide in acidic solutions containing copper, cadmium, zinc and lead ions, were studied in the pH range between 0.5 and 2.0, as well as its influence on the pertraction efficiency, from 0.5 to 0.5 units. Exceeding this interval was not the objective of the study, as the possibility of other ions competing for the precipitation of cations such as hydroxyl and carbonate ions is known.

The selected operating parameters of the installation are presented in Table 3, for experimental test II.

**Figure 12:**
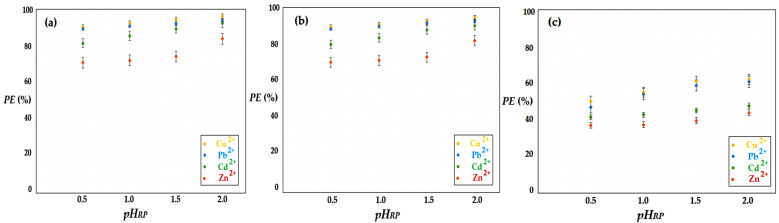
Hydrogen sulfide pertraction efficiency (*PE*%) vs. pH for 20 ppm H_2_S in a gas mixture: nitrogen (**a**), methane (**b**), and carbon dioxide (**c**).

The obtained results (Figure 12) show a slight increase in the pertraction efficiency with the increase in pH, for all studied cations. However, in order to solve the problem of the recuperative separation of metal ions, at the same time as the sequestration of hydrogen sulfide, it is not recommended to increase the pH, as this would mean either the dilution of the source system (acidic solutions from the electronic and electrotechnical industry), or the consumption of reagent neutralization.

In addition, in the case of a gaseous mixture containing carbon dioxide, an increase in pH towards the value of 5 could lead to the consumption of metal ions, which would precipitate as hydroxides or hydroxo-carbonates.

The high value of separation efficiency in the case of gas mixtures in which the matrix was an inert gas (nitrogen, methane) recommends the use of receiving phases containing test metal ions, at the pH and concentration obtained in the recovery processes using the acidic attack of electrotechnical waste.

#### 3.2.3. The Influence of the Hydrogen Sulfide Concentration on Pertraction from a Synthetic Gas Mixture, Using Composite Membranes (Chi/sEPDM/PPy)

The target gas systems for applying the results of the study are those with low gas emissions, containing hydrogen sulfide (inhabited premises in areas with skunks, farms, household waste depots, and water treatment plants). In the experiments on the retention of hydrogen sulfide as metallic sulfides from various gas mixtures, an interval covering the 20–120 ppm range was chosen.

During these experiments, the installation in Figure 3 was operated, using the parameters presented in Table 3, experimental test III.

**Figure 13:**
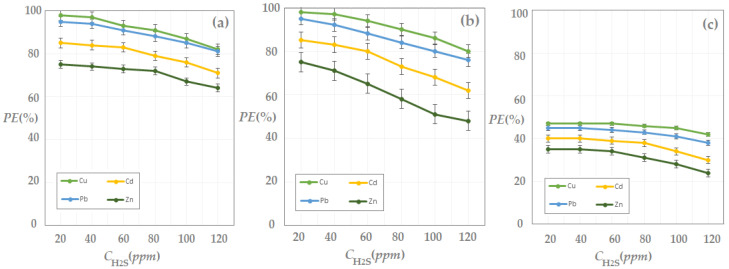
Hydrogen sulfide pertraction efficiency (*PE*%) vs. hydrogen sulfide concentration in gas mixture: nitrogen (**a**); methane (**b**), and carbon dioxide (**c**).

Figure 13 shows the experimental results, which indicate a worsening of the pertraction efficiency with the increase in the concentration of hydrogen sulfide in the feed. Under the specified operating conditions, the pertraction efficiency remained almost constant in the range of 20–60 ppm hydrogen sulfide in the feed.

After this limit, the reduction of the pertraction efficiency was significant, it dropped by more than 30% at a concentration value of 120 ppm H_2_S. From a practical point of view, it is possible to maintain the efficiency of the separation by working in two or more stages of pertraction, but this would require an increase in the membrane surface, and therefore also the cost.

#### 3.2.4. The Influence of the Gas Mixture Flow on Hydrogen Sulfide Pertraction through Chi/sEPDM/PPy Composite Membranes

The flow rate of the source gas mixture constitutes an important parameter of hydrogen sulfide sequestration as sulfides in an acid medium. The range of flow rates of the source gas mixture chosen for the experiments carried out took into account the possibility of improving the quality of reasonable amounts of biogas (domestic installations falling within 2–20 m^3^ of biogas per day), of air in premises (kitchens, bedrooms, offices in agricultural farms), or enclosed hotel spaces in areas with skunks.

The operating parameters of the hydrogen sulfide extraction plant from various gas mixtures are presented in Table 3, experimental test IV.

**Figure 14:**
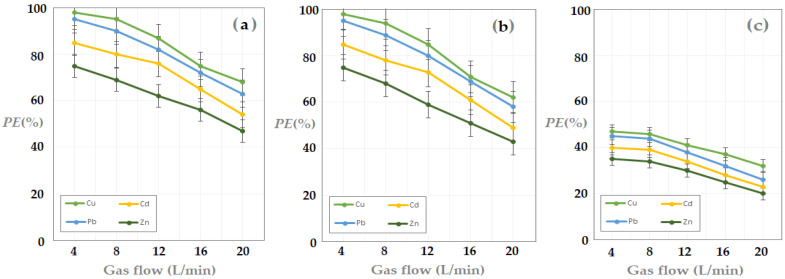
Hydrogen sulfide pertraction efficiency (*PE*%) vs. gas flow mixture: nitrogen (**a**), methane (**b**), and carbon dioxide (**c**).

The efficiency of the hydrogen sulfide extraction from a gaseous mixture (nitrogen, methane, carbon dioxide) decreased as the feed flow increased (Figure 14), for all cases studied.

At a ten-fold increase in the supply flow, the efficiency of the pertraction dropped by almost 40%. The results show that, in order to maintain the efficiency of the installation, the separation surface of the membrane must be increased or it must be operated in several stages of pertraction.

#### 3.2.5. The Proposed Mechanism of the Hydrogen Sulfide Sequestration through Pertraction with Metallic Ions in Acid Media by Chi/sEPDM/PPy–CM from Synthetic Gas Mixtures

The sequestration of hydrogen sulfide as sulfides in an acid medium by pertraction with Chi/sEPDM/PPy–CM was also motivated by the need to capitalize on acidic solutions containing metal ions (Cu^2+^, Zn^2+^, Cd^2+^, Pb^2+^) from waste of the electronic and electrotechnical industry.

There are two main aspects of the proposed technical solution that caused problems when performing the experiments:

- Reticulation of the chitosan (selective membrane material) with which the polypropylene hollow fiber support membrane is impregnated, so that it no longer detaches from the support fiber.
- Optimizing the concentration of metal ions and sulfide ions to ensure the precipitation of metal sulfides and their fixation in the receiving phase.

For the first challenge, which in our previous studies was only partially solved by using sulfonated polyether–ether–ketones [52,56], a resistant polymer with strongly acidic ionic groups sulfonated ethylene-propylene-diene terpolymer was used (sEPDM), which, as shown in Figure 5 and Figure 6, was beneficial.

For the second aspect of the technical solution, the experimental study presented in this paper was carried out. Its theoretical considerations are briefly presented below.

For the partially soluble sulfide of type MS in saturated solution in the presence of the solid phase, the heterogeneous equilibrium can be written as Equation (6):

MS(s) ⇌ MS(aq) ⇌ M^2+^(aq) + S^2^(aq) (6)

The S^2^ anion being basic, depending of the pH, the chemical species HS^−^ and H_2_S are formed following Equilibria couple, Equations (7)–(10):

S^2^ + H_3_O^+^ ⇌ HS^−^ + HOH (7)

with the constant (8):

(8) $$K_{a_{2}}=\frac{\left[S^{2-}\right]\left[H_{3}O^{+}\right]}{\left[{HS}^{-}\right]}=10^{-13}$$

HS^−^ + H_3_O^+^ ⇌ H_2_S + HOH (9)

with the constant (10):

(10) $$K_{a_{1}}=\frac{\left[HS^{-}\right]\left[H_{3}O^{+}\right]}{\left[H_{2}S\right]}$$

Under these conditions, the apparent solubility *S* is calculated using the relation (11) or (12):

*S* = [M^2+^] = [S^2−^] + [HS^−^] + [H_2_S] (11)

(12) $$S=\left[S^{2-}\right](1+\frac{\left[H_{3}O^{+}\right]}{K_{a_{2}}}+\frac{{\left[H_{3}O^{+}\right]}^{2}}{K_{a_{1}}\times K_{a_{2}}})$$

Using the expression for the solubility product K_s_ = [M^2−^][S^2−^] yields the relation (13) (or (14)):

(13) $$S=\sqrt{K_{s}}\times \sqrt{1+\frac{\left[H_{3}O^{+}\right]}{K_{a_{2}}}}+\frac{{\left[H_{3}O^{+}\right]}^{2}}{K_{a_{1}}\times K_{a_{2}}}$$

*S^2^ = K_s_* × (1 + 10^pKa^_2_^−pH^ + 10^pKa^_1_^+pKa^_2_^+2pH^) (14)

which practically can be used to demonstrate the influence of both the metal ion concentration and the pH on the efficiency of the pertraction in well-defined working conditions.

**Figure 15:**
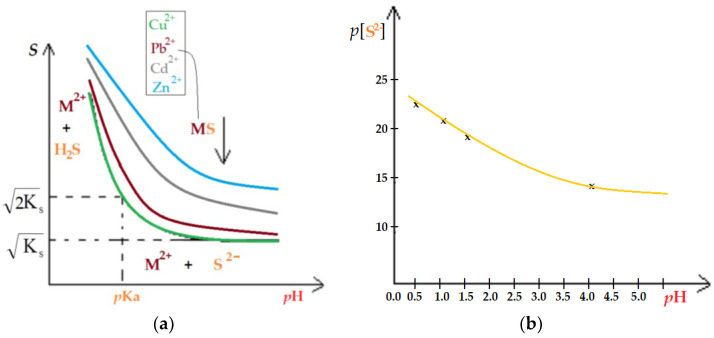
Diagrams of the sulfide metals solubility (*S*) vs. pH (**a**); and pS^2−^ vs. pH (within the range of interest) (**b**).

Figure 15 shows characteristic diagrams that can explain the formation of sulfides of the metal ions considered.

Thus, when the pH decreases, the stability of sulfides is in the order: Cu^2+^ > Pb^2+^ > Cd^2+^ >> Zn^2+^. Practically, at a given acidic pH, copper sulfide precipitates first, then lead, cadmium, and finally zinc.

In the case of a multicomponent solution, in the proposed membrane system, the sulfides of metal ions can be used separately, simultaneously with the sequestration of hydrogen sulfide.

**Figure 16:**
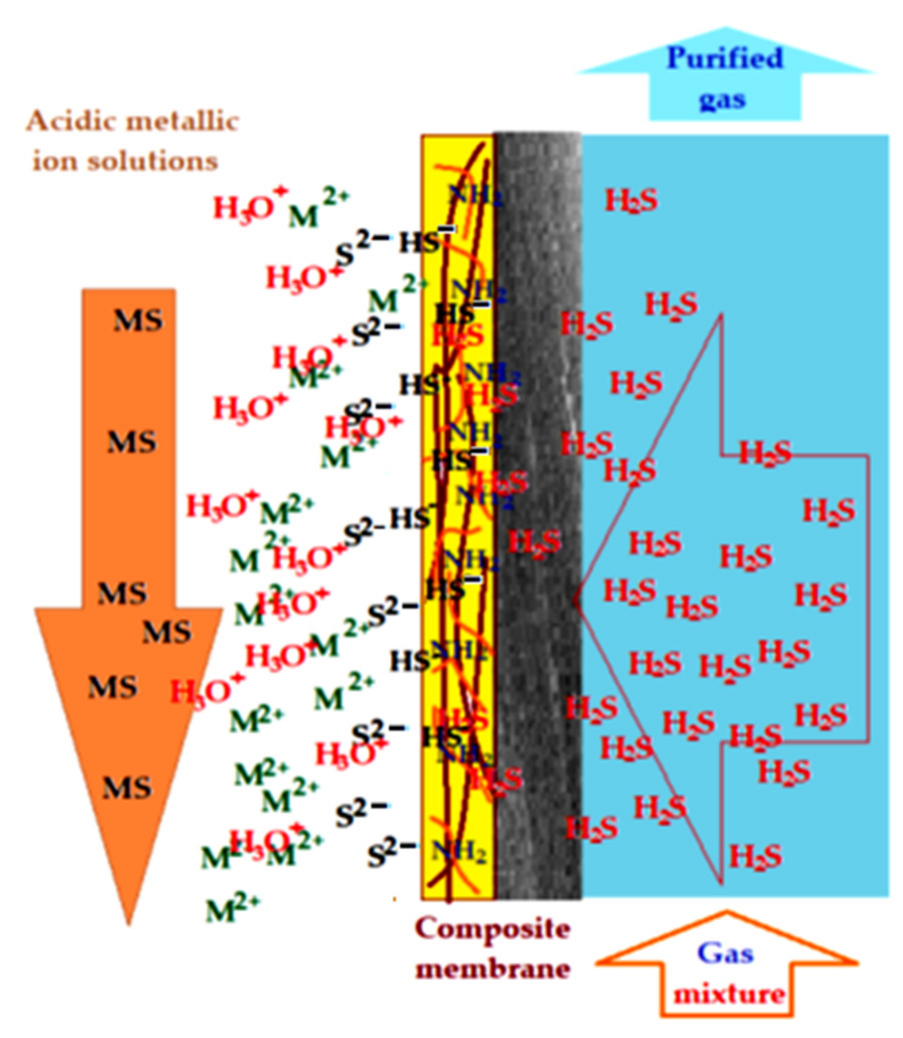
Schematic mechanism of the hydrogen sulfide sequestration by pertraction with Chi/sEPDM/PPy–CM in acid medium containing metal ions, from synthetic gas mixtures.

The most likely mechanism of the sequestration of hydrogen sulfide as sulfides in an acid medium by pertraction with Chi/sEPDM/PPy–CM using acidic solutions containing metal ions (Cu^2+^, Zn^2+^, Cd^2+^, Pb^2+^) is schematized in Figure 16.

The sequestration of hydrogen sulfide by pertraction with Chi/sEPDM/PPy–CM takes place in several stages:

- Diffusion of gases from the source phase through polypropylene hollow fiber membrane support.
- Hydrogen sulfide concentration in the selective chitosan layer of the composite membrane through a solubilization–extraction mechanism in the solid phase and diffusion towards the interface with the receiving acid solution containing metal ions.
- Solubilization of hydrogen sulfide in the receiving phase.
- Immobilization of hydrogen sulfide through precipitation as metallic sulfide in the receiving phase.

The described stages explain both the better results of the extraction of hydrogen sulfide from gas mixtures based on nitrogen and methane, as well as the decrease in the efficiency of the extraction of hydrogen sulfide from the mixture based on carbon dioxide, which is in competition with hydrogen sulfide in the solubilization–extraction stage in the active layer of chitosan. The selectivity of the process is provided by the fact that, in conditions of pronounced acidity in the receiving phase, only the sulfides of the considered metals are formed and not their carbonates or hydroxides. The solubility products of the considered metal ion pairs are in full agreement with the results obtained in the experiments with each individual ion.

#### 3.2.6. Practical Aspects and Application Perspectives

The proposed hydrogen sulfide sequestration system using Chi/sEPDM/PPy–CM transport and separation, followed by precipitation as partially soluble sulfides in an acid medium is in the research phase, which allows the continuation of the study regarding the optimization and technical–economic evaluation.

Although the range of efficiency of hydrogen sulfide separation from the considered gaseous matrices is quite narrow (20–60 ppm), it exceeds the usual concentrations of the target gases treated (air from domestic premises in areas exposed to pollution, natural methane gas, biogas).The decreases in efficiency when the concentration was increased above about 60 ppm H_2_S in the feed was caused by the solubility product reaching the boundary layer of the membrane in contact with the acid receiving solution of metal ions and the precipitation of sulfides on the surface of the membrane. This aspect was especially noted for higher concentrations of hydrogen sulfide in the feed and simultaneously of metal ions in the receiving phase. For this limit situation, a study of unclogging of the membrane surface based on the effect of cavitation, mechanical vibrations, or ultrasound has been provided. The hydrodynamic cavitation method for membrane unclogging involves the use of a piston pump that feeds a set of converging nozzles with a neck diameter of 0.2 mm, which allows the development of high cutting forces specific to hydrodynamic cavitation [73].

**Figure 17:**
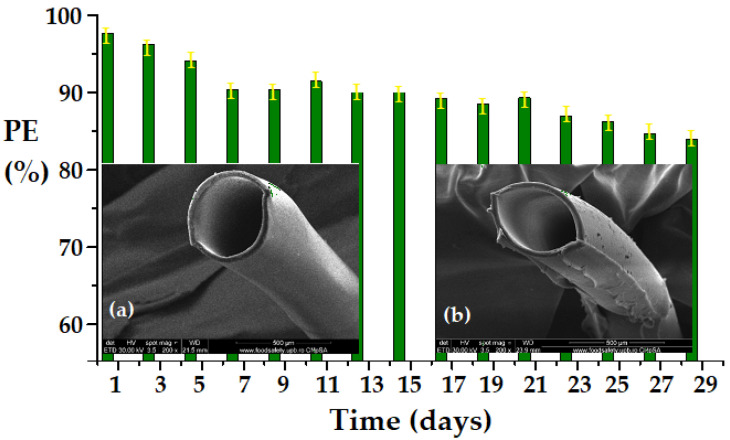
Pertraction efficiency (PE%) of Chi/sEPDM/PPy–CM during four weeks of operation in the system (30 ppm H_2_S, in nitrogen, with a feed flow of 4 L/min and a receiving phase with 10^−2^ mol/L cadmium ions at pH = 2): (**a**) SEM image of the membrane at the beginning of the experiment; (**b**) SEM image of the membrane at the end of the experiment.

From the point of view of the stability over time of the separation efficiency, it was found that the traces of hydrogen sulfide can be removed by sequestration with metal ions, in acid media, as partially soluble sulfides, with an efficiency of over 80% (Figure 17).

The evolution of the system over a period of four weeks, and the appearance of the membrane before (Figure 17a) and at the end of the period (Figure 17b), was evaluated in a test experiment with a source phase of 30 ppm H_2_S in a nitrogen matrix, a feed flow of 4 L/min, and a receiving phase containing 10^−2^ mol/L cadmium ions at pH = 2. The results illustrated in Figure 17 show a slight change over time of the composite layer of the membrane (Figure 17b), and at the end of the experiment the pertraction efficiency did not drop below 82%. On the tenth and twentieth days of the experiment, the pertraction module was mechanically vibrated, which caused a slight increase in the separation efficiency (most likely due to the detachment of the precipitate from the fibers).

With regard to the circuit of the source phase “through” or “between” the fibers, the choice of the working process took into account that the area where the precipitate appears should be accessible.

However, it is possible to opt for the introduction of the impure gas between the fibers, and the acid solution of metal ions through the fibers, if the recovery of the precipitate that accumulates in the fibers is performed together with the fiber. Thus, in the case of the sequestration of hydrogen sulfide as cadmium sulfide, at the end of the separation process, the fiber that contains cadmium sulfide can be processed as such, to obtain photoluminescent materials with multiple applications.

Regarding the competitive permeation of the components of the feed gas mixture, it is known that each component of the Chi/sEPDM/PPy–CM composite membrane makes a contribution: the polypropylene support fiber provides a large contact surface and physicochemical resistance, chitosan improves the selectivity, and sEPDM was chosen as an excellent adhesion material for fibers and crosslinking for chitosan. The relatively low molecular weight and strongly acid medium would have led to the dissolution of the chitosan layer.

In our case, the chosen gaseous systems assume trace amounts of hydrogen sulfide, and the choice of chitosan favors its permeation, followed by sequestration as sulfides. The installation and the working mode considered a parallel permeation of the other gases, and therefore a loop was provided to separate them from the pertraction module. Although the permeation of nitrogen or methane does not significantly affect the efficiency of the hydrogen sulfide removal process, in the case of carbon dioxide, the effects are significant and unfavorable.

In the case of gaseous mixtures that also contain other acid gases (SOx and NOx), some remarks can be made regarding the applicability and prospects of the proposed process. First of all, sulfides and nitrates will not be captured in the acid solution, because the salts of the chosen metal ions are soluble. However, the Chi/sEPDM/PPy–CM composite membrane will be affected by the competitive interaction with acid species other than hydrogen sulfide, and the separation efficiency is likely to decrease.

Thus, in the case of a complex mixture of acid gases in an inert matrix (nitrogen, methane), the sequestration of hydrogen sulfide in a solution of metal ions in a slightly basic medium (for example ammonia) would be preferred.

The choice of sequestration with metal ions in acid solutions has as its objective the integration of the process on complex urban waste recycling platforms, where we encounter both the production of biogas (with traces of hydrogen sulfide), and acid solutions containing metal ions (Cu^2+^, Cd^2+^, Pb^2+^ and/or Zn^2+^) from the recycling of electronic and electrotechnical waste (urban waste processing platforms).

## 4. Conclusions

This paper presents the preparation and characterization of composite membranes based on chitosan (Chi), sulfonated ethylene–propylene–diene terpolymer (sEPDM), and polypropylene (PPy) designed for hydrogen sulfide sequestration. The composite membranes were morphologically and structurally characterized using scanning electron microscopy (SEM), Fourier transform infrared spectroscopy (FTIR), energy-dispersive spectroscopy analysis (EDAX), thermal analysis (TG, DSC), thermal analysis coupled with chromatography, and infrared analysis. They were also evaluated from the point of view of the pertraction performance obtained for sequestration of the hydrogen sulfide in acid media solutions containing metal ions (Cu^2+^, Cd^2+^, Pb^2+^, and/or Zn^2+^).

The operational parameters of the pertraction were the pH, pM, matrix gas flow rate, and the composition. The results of the extraction from synthetic gas mixtures (nitrogen, methane, carbon dioxide) indicated an efficient removal of hydrogen sulfide through the prepared composite membranes and its immobilization as sulfides. Sequestration and recuperative separation as sulfides from an acid medium of hydrogen sulfide reached 96%, decreasing in the order: CuS > PbS > CdS > ZnS.

The efficiency of hydrogen sulfide removal from gas mixtures decreased in the order nitrogen > methane > carbon dioxide. The realized recuperative separation method can be, at the same time, a way to valorize acid solutions in the processing of Cu–Zn or Pb–Cd type waste, resulting from the electronic and electrotechnical industries.

The system parameters leading to the highest pertraction efficiencies, regardless of the cation in the receiving solution, were: source phase 30–60 ppm H_2_S in nitrogen or methane, with the feed rate of 2–8 L/min and a receiving phase with of 500 mL/min, 10^−2^ mol/L metal ions, at pH = 2.

This paper contributes practical aspects of the application and perspectives on a hydrogen sulfide sequestration system using Chi/sEPDM/PPy–CM.

## Figures and Tables

**Table 1:** The hydrogen sulfide removal processes and specific characteristics and applications.

Hydrogen Sulfide Removal Processes	Characteristics	Efficiency (%)	Specific Applications	Refs.
Precipitation	pH = 2–4, metal ions solution	>90%	metals recovery from mine water	[11]
Adsorption	200–1500 ppm H_2_S, 200–1200 mL/min	>70%	adsorption with biochar from synthetic mixture	[12]
Absorption (Scrubbing)	natural or synthetic zeolites, activated carbons, and metal oxides	80–95%	H_2_S capture	[13]
Chemosorption	iron oxides and polymer composites	>95%	biogas purification	[14]
Extraction	various systems	>80%	H_2_S removing	[15]
Oxidative degradation	Fenton reagents	depends on applications	H_2_S recovery as sulfuric acid or elemental sulfur	[16]
Electrochemical degradation	membrane and electrochemical systems	>90%	thermochemical processing of contaminated biomass	[17]
Photo-catalytic degradation	TiO_2_ and non-TiO_2_ based catalysts	depends on applications	hydrogen production and environmental remediation	[18]
Catalytic degradation	ZnO–MgO/activated carbon	113.4 mg/g–96.5 mg/g		[19]
Biological degradation	2000 ppm H_2_S, low pH, pilot scale	>97%	H_2_S green removal from a gas mixture	[20]
Polymeric membranes	various conditions	depends on applications	natural gas purification	[21]
Emulsion liquid membrane	high salinity wastewater	>97%	H_2_S removal from sea water	[22]
Supported liquid membrane	ionic liquid membrane on inorganic support	depends on applications	acid gases separation from gas mixtures	[23,24]
Membrane contactor	various porous membrane contactors	depends on applications	gases removal or recovery	[25,26]
Hollow fiber contactor	polydimethylsiloxane	98% H_2_S and 59% CO_2_	biogas purification	[27]
Hollow fiber contactor	mono-ethanolamine	>95%	H_2_S removal from gas mixture	[28]
Hybrid processes	membrane separation and oxidation	97% removing, 74% conversion	H_2_S removal and degradation	[29]

**Table 2:** The characteristics of the used polymers.

Polymers	Name and Symbol	Molar Mass (g/mol)	Solubility in Water (g/L)	pKa
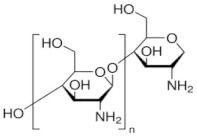	Chitosan (Chi)	1526.5	soluble in acid media (0.5 M HCl: 50 mg/mL)	6.2 to 7.0
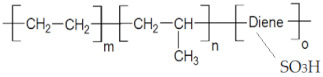	sulfonated ethylene– propylene–diene terpolymer (sEPDM)	3500–5500	soluble in toluene	1.9 to 2.2

**Table 3:** Membrane system parameters used in the study of the H_2_S pertraction.

Experimental Test	Membrane	Hydrogen Sulfide Feed	Flow Rate of Source Phase	Receiving Phase	Flow Rate of Receiving Phase	pM	Pertraction Experiment
I	Chi/sEPDM/PPy	20 ppm	10 L/min	5.0 L, pH 0.5	100 mL/min	3–7	5 h
II	Chi/sEPDM/PPy	20 ppm	2 L/min	pH 0.5–2.0	300 mL/min	3	5 h
III	Chi/sEPDM/PPy	20–120 ppm	4 L/min	pH 1	500 mL/min	3	5 h
IV	Chi/sEPDM/PPy	40 ppm	2–20 L/min	pH 1	500 mL/min	3	5 h

## Data Availability

All data are contained within the article.

## Supplementary Materials

The following supporting information can be downloaded at: https://www.mdpi.com/article/10.3390/membranes13030350/s1. Figure S1: Schematic presentation of the hydrogen sulfide undesired characteristics and the processes to prevent and/or combat them, Figure S2: The evolution of traces of substances depending on the temperature (°C) at specific wave numbers.

## Abbreviations

Chi	Chitosan
sEPDM	Sulfonated ethylene–propylene–diene terpolymer
PPy	Polypropylene
Chi/sEPDM/PPy–CM	Chitosan/sulfonated ethylene–propylene–diene terpolymer/polypropylene composite membrane
SEM	Scanning Electron Microscopy
HR–SEM	High Resolution Scanning Electron Microscopy
FTIR	Fourier Transform Infra-Red spectroscopy
EDAX	Energy-Dispersive Spectroscopy Analysis
TA	Thermal Analysis
TG	Thermo-Gravimetric
DSC	Differential Scanning Calorimetry
GC	Gas Chromatography
UV–Vis	Ultraviolet-Visible Spectrophotometry
AAS	Atomic Absorption Spectrometry
MIR	Fourier Transform Infra-Red Microscopy
PE	Pertraction Efficiency
pH	-log [H_3_O]
pH	-log [M^2+^]
