# Supplementary material for: Hydrogen Sulphide Sequestration with Metallic Ions in Acidic Media Based on Chitosan/sEPDM/Polypropylene Composites Hollow Fiber Membranes System
Source: Membranes (Basel). 2023 Mar 17;13(3):350. doi: 10.3390/membranes13030350 (PMC10057485; doi:10.3390/membranes13030350)
Supplement: Supplementary file 1 [file membranes-13-00350-s001.zip › membranes-2223212-supplementary.pdf]

membranes

Supplementary Materials

Hydrogen Sulphide Sequestration with Metallic Ions in Acidic Media Based on Chitosan/sEPDM/Polypropylene Composites Hollow Fiber Membranes System

Dumitru Pașcu [1], Aurelia Cristina Nechifor [1], Vlad-Alexandru Grosu [2,*], Ovidiu Cristian Oprea [3], Szidonia-Katalin Tanczos [4], Geani Teodor Man [1], Florina Dumitru [3], Alexandra Raluca Grosu [1] and Gheorghe Nechifor [1,*]

[1] Analytical Chemistry and Environmental Engineering Department, University Politehnica of Bucharest, 011061 Bucharest, Romania
[2] Department of Electronic Technology and Reliability, Faculty of Electronics, Telecommunications and Information Technology, University Politehnica of Bucharest, 061071 Bucharest, Romania
[3] Department of Inorganic Chemistry, Physical Chemistry and Electrochemistry, University Politehnica of Bucharest, 011061 Bucharest, Romania
[4] Department of Bioengineering, University Sapientia of Miercurea-Ciuc, 500104 Miercurea-Ciuc, Romania
* Correspondence: vlad.grosu@upb.ro (V.-A.G.); ghnechifor@gmail.com (G.H.)

Abstract: This paper presents the preparation and characterization of composite membranes based on chitosan (Chi), sulfonated ethylene–propylene–diene terpolymer (sEPDM), and polypropylene (PPy), and designed to capture hydrogen sulfide. The Chi/sEPDM/PPy composite membranes were prepared through controlled evaporation of a toluene dispersion layer of Chi:sEPDM 1;1, *w/w*, deposited by immersion and under a slight vacuum (100 mmHg) on a PPy hollow fiber support. The composite membranes were characterized morphologically, structurally, and thermally, but also from the point of view of their performance in the process of hydrogen sulfide sequestration in an acidic media solution with metallic ion content (Cu^{2+}, Cd^{2+}, Pb^{2+}, and/or Zn^{2+}). The operational parameters of the pertraction were the pH, pM, matrix gas flow rate, and composition. The results of pertraction from synthetic gases mixture (nitrogen, methane, carbon dioxide) indicated an efficient removal of hydrogen sulfide through the prepared composite membranes, as well as its immobilization as sulfides. The sequestration and the recuperative separation, as sulfides from an acid medium, of the hydrogen sulfide reached up to 96%, decreasing in the order: CuS > PbS > CdS > ZnS.

Keywords: hydrogen sulphide sequestration; composite membranes; chitosan; sEPDM; polypropylene hollow fiber membrane; electronics waste

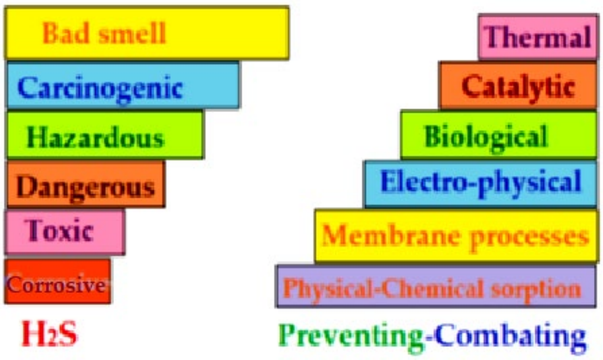

Figure S1. Schematic presentation of the hydrogen sulfide undesired characteristics and the processes to prevent and/or combat them.

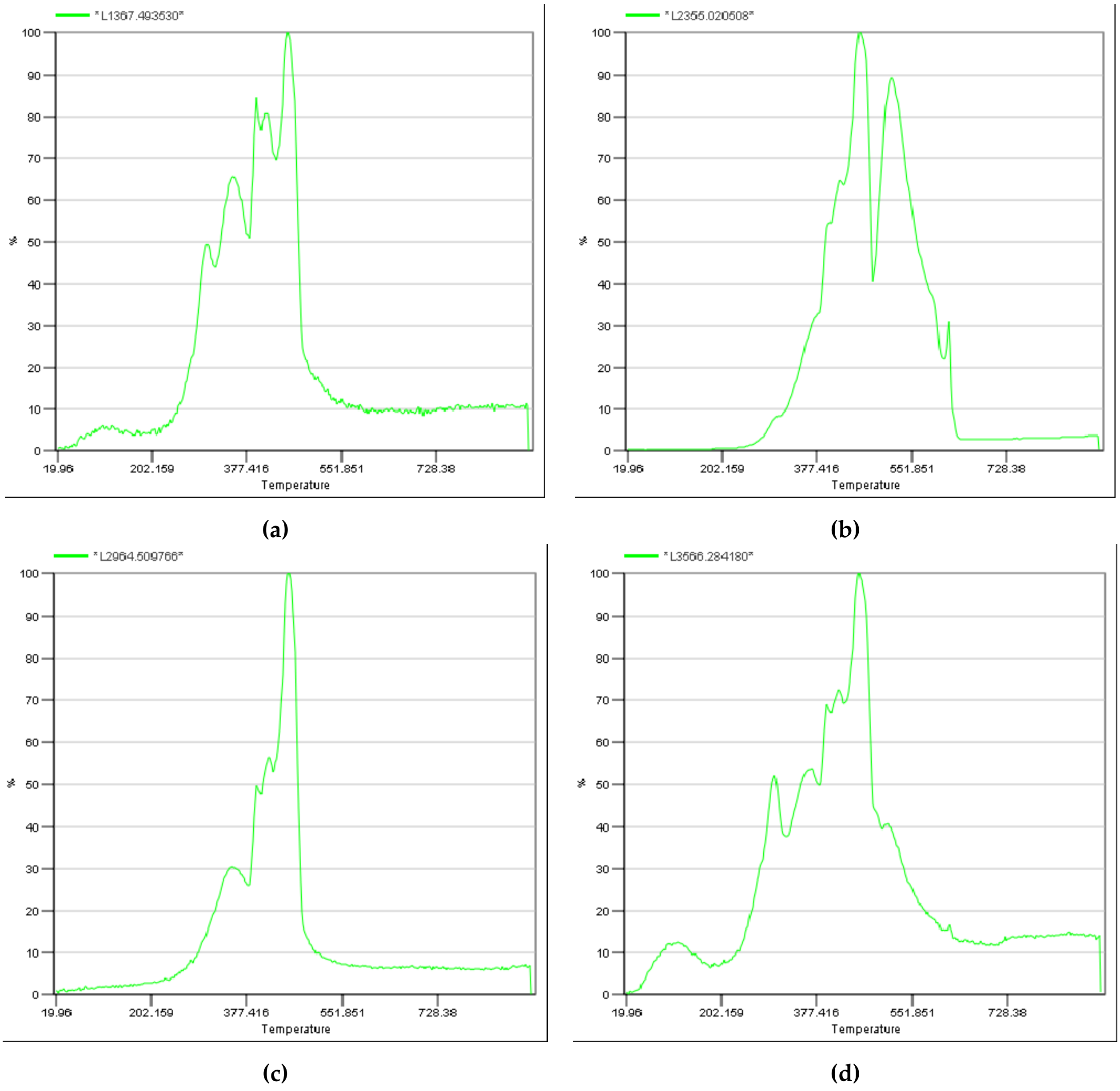

Figure S2. The evolution of traces of substances depending on the temperature (°C) at specific wave numbers: (**a**) SO_2 (1367 cm^{-1}); (**b**) CO_2 (2355 cm^{-1}); (**c**) hydrocarbons (2964 cm^{-1}); and (**d**) H_2O (3566 cm^{-1}).
